# Leptin promoter methylation in female patients with painful multisomatoform disorder and chronic widespread pain

**DOI:** 10.1186/s13148-022-01235-5

**Published:** 2022-01-21

**Authors:** Johannes Achenbach, Mathias Rhein, Alexander Glahn, Helge Frieling, Matthias Karst

**Affiliations:** 1 Department of Anesthesiology and Intensive Care Medicine, Nordstadt Krankenhaus Hannover, Haltenhoffstr. 41, 30167 Hannover, Germany; 2 Department of Anesthesiology and Intensive Care Medicine, Pain Clinic, Hannover Medical School, Carl-Neuberg-Str. 1, 30625 Hannover, Germany; 3 Laboratory for Molecular Neuroscience, Department of Psychiatry, Social Psychiatry and Psychotherapy, Hannover Medical School, Carl-Neuberg-Str. 1, 30625 Hannover, Germany

**Keywords:** Leptin, Methylation, Multisomatoform disorder, Fibromyalgia, Chronic pain

## Abstract

**Background:**

Different functional somatic syndromes (FSS), fibromyalgia (FMS) and other unexplained painful conditions share many common clinical traits and are characterized by troubling and functionally disabling somatic symptoms. Chronic pain is most frequently reported and at the center of patients’ level of disease burden. The construct of multisomatoform disorder (MSD) allows to subsume severely impaired patients suffering from FSS, FMS and other unexplained painful conditions to be examined for common underlying processes. Altered leptin levels and a pathological response of the HPA-axis as a result of chronic stress and childhood trauma have been suggested as one of the driving factors of disease development and severity. Previous studies have demonstrated that methylation of the leptin promoter can play a regulatory role in addiction. In this study, we hypothesized that methylation of the leptin promoter is influenced by the degree of childhood traumatization and differs between patients with MSD and controls. A cohort of 151 patients with MSD and 149 matched healthy volunteers were evaluated using clinical and psychometric assessment while methylation level analysis of the leptin promoter was performed using DNA isolated from whole blood.

**Results:**

In female controls, we found CpG C-167 to be negatively correlated with leptin levels, whereas in female patients CpG C-289, C-255, C-193, C-167 and methylation cluster (C-291 to C-167) at putative bindings sites for transcription factors Sp1 and c/EBPalpha were negatively correlated with leptin levels. Methylation levels were significantly lower in female patients CpG C-289 compared with controls. When looking at female patients with chronic widespread pain methylation levels were significantly lower at CpG C-289, C-255 and methylation cluster (C-291 to C-167).

**Conclusion:**

Our findings support the hypothesis that epigenetic regulation of leptin plays a role in the regulation of leptin levels in patients with MSD. This effect is more pronounced in patients with chronic widespread pain.

**Supplementary Information:**

The online version contains supplementary material available at 10.1186/s13148-022-01235-5.

## Background

In patients presenting with painful symptoms often a sufficient underlying explanation in terms of a somatic diagnosis cannot be found. In these cases, the chronic pain can be characterized as the leading symptom of a functional somatic syndrome (FSS) such as fibromyalgia (FMS) or somatoform pain disorder. In such syndromes, functionally disabling and bothersome physical symptoms are also frequently present. This constellation of symptoms is also present in multisomatoform disorder (MSD) [1, 2] which is a diagnostic construct to better characterize these patients across different somatic and psychological specialties [1, 3]. A diagnosis of MSD can be made in the presence of more than three currently distressing physical symptoms in addition to a long (greater than 2 years) history of somatization. The prevalence of MSD is 8% and thus posts a relevant disease burden [3] The pathophysiology of functional somatic syndromes, fibromyalgia, and MSD is incompletely understood but a complex interplay of biographic, environmental, genetic, and epigenetic factors influencing allostasis seems likely [4, 5], especially as the similarity in symptoms and patients suggest common mechanisms which lends validity to the construct of MSD. In a population-based twin study, genetic influences have been shown to play a role especially in painful FSS, whereas inconsistent results suggest a role of single nucleotide polymorphisms (SNPs) of serotonergic and dopaminergic genes [6–8]. Our group recently demonstrated common sensory alterations through quantitative sensory testing in patients with MSD [9] similar to those found in patients with fibromyalgia (FMS) [10, 11]. In this context, the construct of chronic widespread pain is of particular interest. Since its systematic introduction as part of the diagnostic criteria for FMS in 1990 [12] numerous studies have included patients with CWP not fulfilling criteria for FMS. However, different interpretations of the criteria as well as adaptations over time [13, 14] have made comparisons not straightforward [15]. DNA methylation describes a modification through covalent binding of a methyl group to cytosine residues that are followed by guanine nucleotide in the DNA strand (CpG Island). This has among others been shown to be influenced in a model of early stress through reduced neonatal maternal care in rodent models [16–18] as well as in chronic pain states [19, 20]. We could also demonstrate the influence of transient receptor potential ankyrin 1 (TRPA1) receptor promoter methylation on heat and pressure pain thresholds which was significantly influenced by the level of childhood traumatization [21].

Lastly, the complex interplay between obesity in chronic pain states as well as FMS, leptin and the HPA-axis has been investigated with growing interest by the scientific community [22], whereas obesity is a common comorbidity in FMS and has also been shown to increase symptom severity [23–26], Leptin levels in relation to painful conditions have been found to be either unaltered [27], elevated [28–31] or reduced [32] compared with controls. Leptin is a 16 kDa protein predominantly secreted by adipose tissue or in the brain [33, 34]. Its main function lies in the regulation of energy homeostasis and conveying a feeling of satiety [35–37]. It has also been shown to have an inhibitory function on the HPA-axis [38]. In a reverse manner, however, its synthesis is stimulated by cortisol in adipose tissue [39]. Additionally, leptin has been demonstrated to play a role in the pathophysiology of neuropathic pain [40–42] . The expression of leptin has been previously shown to be influenced by epigenetic mechanisms, namely hypomethylation in the promoter region at binding sites for Sp1 and C/EBPalpha [43–46] which typically act as activators of gene expression.

Our group recently demonstrated in the current patient collective a distinct alteration of the neuroendocrine profile of patients with MSD (publication under review) with a significantly higher level of leptin and lower levels of cortisol in female patients compared with controls. We, therefore, hypothesized that in patients with MSD the difference in measured leptin levels is influenced by alterations in leptin promoter methylation due to the influence of childhood trauma.

## Materials and methods

### Subjects

Participants in this study have been previously evaluated with regards to the presence of SNPs of different genes [6–8], the presence of sensory alterations using standardized quantitative sensory testing as well as methylation status of the TRPA1 promoter [9, 21]. Altogether, 151 MSD patients and 149 healthy controls were included in the study. Patients were recruited through the outpatient pain clinic of the Hannover Medical School, Hannover, Germany, and the Clinic for Psychosomatic Medicine and Psychotherapy of the Hannover Medical School over a period of 12 months. Additional patients were contacted through local fibromyalgia support groups while healthy age- and gender-matched participants without physical pain were included in the control group. Exact records of the place of recruitment were not kept; most patients however were partaking in regular treatments at Hannover Medical School. Severe somatic or psychiatric conditions were excluded through expert clinician assessment while psychometric evaluations through questionnaires were also performed. All patients’ chief complaint was chronic widespread pain. Diagnosis of MSD was supported by means of a modified interview of the somatoform disorders section of the Structured Clinical Interview for the Diagnostic and Statistical Manual of Mental Disorder IV (DSM-IV) (SCID) as well as the German version of the 36-item Short Form 36 (SF-36) questionnaire, i.e., the Physical Component Summary score needed to be ≤ 40 as sign of strong psychophysiological strain [1, 2, 6–8, 47]. The presence of chronic widespread pain fulfilling the strict criteria of pain present in three out of four body quadrants in addition to axial pain [15] was systematically assessed by a 34-item pain localization questionnaire.

Exclusion criteria were defined as age < 18 years, insufficient German language ability, insufficient cognitive abilities, severe and chronic somatic diseases (e.g., severe heart failure, encephalitis disseminata, dementia), and severe comorbid mental disorders which cause major impairment of social functioning (e.g., schizophrenia, severe mood disorders, personality disorders, substance abuse) as previously described [6–8]. Psychometric questionnaires are beyond the scope of the current manuscript.

Blood samples were collected and used for DNA extraction, laboratory, and epigenetic analysis [48, 49]. In all investigations, the revised Declaration of Helsinki in 2000 (Edinburgh, 52. general meeting) was adhered to and there was approval by the Ethical Committee of Hannover Medical School (study protocol number 4757). All subjects gave informed consent for blood sampling, genotyping, and clinical measurements [6–8].

### Determination of Leptin levels

A radioimmunoassay was performed using the human leptin RIA kit (LINCO Research, St. Charles, Missouri, USA). Blood was collected between 8.00 and 9.00 am for each participant to be in keeping with the circadian rhythm of hormone release of the HPA-axis. EDTA vials (4 ml) and Serum vials (5 ml) were used (S-Monovette, Sarstedt). Measurements were performed through the Department of Endocrinology of the Hannover Medical School (MHH).

### DNA Isolation

Blood was collected from each subject using two 4-mL EDTA tubes that were then stored at − 80° until extraction. Genomic DNA from patients and controls was extracted using a standard high-salt extraction method. A small subset of DNA samples was isolated by using a commercially available DNA isolation kit (QiAamp® blood kit, Qiagen, Hilden, Germany) according to the manufacturer’s instructions.

### Determination of methylation rates

DNA was bisulfite-converted using the Epitect conversion kit (Qiagen, Hilden, Germany) according to manufacturer recommendations.

Bisulfite-converted DNA was used for PCR amplification using specific primer sets (see Additional file 1: Table S1) in a Touchdown PCR approach [50]. Resulting amplicons were subjected to linear sequencing PCR using BigDye Terminator according to manufacturer instructions (ABI Life Technologies, Grand Island, USA). For Sequence cleanup prior to sequencing we used AMPure beads on a Biomek NxP liquid handling platform (Beckman Coulter, Brea, USA). Purified reactions were sequenced using a 3500xl 24 capillary Sequencer (ABI Life Technologies, Grand Island, USA).

CpG position is provided in relation to the transcriptional start site located at GRCh38:7:128241278 according to ENSEMBL gene accession # ENSG00000174697. All reported locations are in the proximal promoter upstream of the gene locus. Sequence analysis and determination of methylation rates for each CpG site were conducted using the Epigenetic Sequencing Methylation analysis software [51]. The methylation rate of each CpG site per subject was estimated by determining the ratio between normalized peak values of cytosine and thymine.

### Quality control

Raw sequences were checked for quality and integrity by using the Sequence Scanner 2 Software (ABI Life Technologies, Grand Island, USA) and alignment in Geneious 11 (Biomatters, Auckland, New Zealand).

The resulting values were processed further if 95% of the CpGs of each specimen and 95% of the respective CpG position were available.

We successfully measured other genes in this collective (TRPA1) [21] as well as unpublished data. The overall variance of measured results for TRPA1 and other genes was very low indicating a high level of precision of the collected data.

### Prediction of transcription factor binding sites

Potential binding sites for transcription factors (TFs) were predicted using Geneious 11 (Biomatters, Auckland, New Zealand) allowing for 1 mismatch base. The findings were confirmed using the Alggen Promo tool (http://alggen.lsi.upc.es/cgi-bin/promo_v3/promo/promoinit.cgi?dirDB=TF_8.3) on the same sequence. Both tools access the freely available resources at the Transfac public database: (http://gene-regulation.com/cgi-bin/pub/databases/transfac/search.cgi) [52].

### Statistical analysis

All statistical calculations were performed using the Statistical Package for the Social Sciences Version 26 (SPSS, IBM, Armonk, NY). We used GraphPad Prism for Mac Version 9 for data illustration (Graphpad Software Inc, La Jolla, CA). Sequence Scanner v1.0 software (ABI Life Technologies) was used to assess sequence quality. After sample quality estimation 151 Patients and 149 controls were used for data analysis. CpG sites were measured successfully without need for exclusion from analysis. Distribution of data was checked according to Shapiro–Wilk. For normally distributed data parametric tests were chosen, in all other instances nonparametric tests were used. Pearson correlations were used to characterize association of methylation with serum leptin levels. Differences between patients and controls were assessed using a two-sided t test for independent samples. Adjustment for multiple comparisons was not made as comparisons were preplanned before the study was commenced. Equality of variance was determined automatically using the Levene test. Interpretation of the results was conducted accordingly. Results are given as mean and standard deviation.

## Results

Diagnostic criteria for MSD according to the Diagnostic and Statistical Manual of Mental Disorder-IV (DSM-IV) were fulfilled by all patients. As previously reported, there were no differences between gender and age (*p* > 0.05) (control group: mean age, 52.1 ± 9.9 years; 73% women and 27% men; MSD group: mean age, 54.4 ± 10.1 years; 82% women and 18% men) [6–9, 21]. As expected the physical component summary score of the SF-36 demonstrated a significant difference between patients and controls (28.75 ± 7.81 vs 54.0 ± 5.74) (*p* < 0.0001). The sample size of male participants proved too small to provide satisfactory explanatory power. At the same time, no significant findings could be demonstrated so that further investigation focused mainly on female study participants. Leptin measurements were obtained in 244 participants (129 female controls, 91 female patients as well as 12 male patients and controls each (data submitted for publication).

### Methylation

**Fig. 1:**
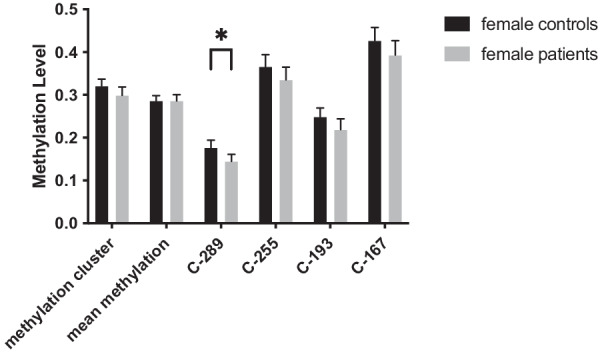
Methylation levels of most relevant CpGs comparing female patients and female controls. Data represented as mean + 95% CI. There was a significant difference observed only at CpG C-289

Leptin levels and methylation status at the following CpGs were negatively correlated: in female controls at C-167 (rp = − 0.205, *p* = 0.046) and in female patients at C-289 (rp = − 0.232, *p* = 0.047), C-255 (rp = − 0.242, *p* = 0.038), C-193 (rp = − 0.294, *p* = 0.022), C-167 (rp = − 0.242, *p* = 0.043) and the mean methylation at the cluster with binding sites for Sp1, c/EBPalpha and CREB (C-291 til C-167) (rp = − 0.239, *p* = 0.039). The observed correlations affected CpGs that were in close proximity to one another and have been previously shown to have particular relevance as binding motifs for Sp1, c/EBPalpha and CREB which are well known to be involved in the regulation of leptin expression. We, therefore, decided to further characterize only these highly thematic CpGs. There was a significant difference in the methylation levels of CpG C-289 between female patients (0.1449 ± 0.9554) and controls (0.1766 ± 0.1000), *t*(211) = 2.366, *p* = 0.019 (see Fig. 1 for most relevant CpGs). For a graphical representation of the methylation level at each individual CpG see Additional file 2: Fig. S1.

**Fig. 2:**
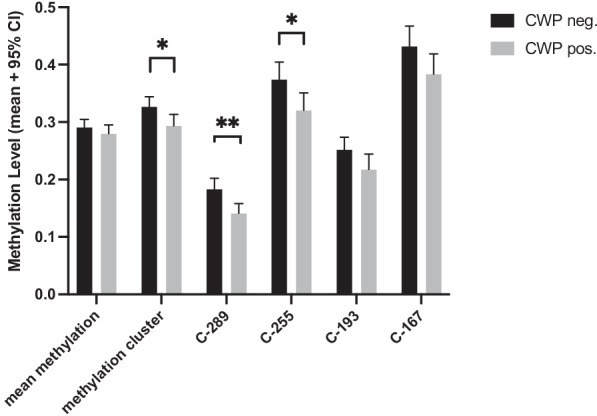
Methylation levels of most relevant CpGs comparing female patients fulfilling strict criteria for chronic widespread pain and female controls. Data represented as mean + 95% CI. There was a significant difference observed CpG C-289, C-255 and methylation cluster

Significant differences between female patients and controls despite significant correlation with leptin levels could surprisingly only be found at CpG C-289. To focus on patients with the highest pain burden, we re-examined a subset of female patients (120/138) fulfilling the strict criteria for chronic widespread pain (pain in three out of four quadrants as well as axial pain). Incomplete data to determine pain distribution were present in 5 female controls and 10 female patients. Significant differences were observed at CpG C-289 (*t*(182) = 2.990, *p* = 0.003), C-255 (*t*(182) = 2.202, *p* = 0.029) and methylation cluster (*t*(183) = 2.228, *p* = 0.024). A graphical representation is given in Fig. 2, whereas exact methylation levels are given in Table 1.

**Table 1:** Mean methylation levels of female patients with chronic widespread pain (CWP) and female controls without CWP; SD: standard deviation

	CWP negative		CWP positive	
	Mean	SD ±	Mean	SD ±
Mean methylation	.2911	.0723	.2800	.0828
Methylation cluster	.3270	.0898	.2935	.1106
C-289	.1835	.0987	.1414	.0920
C-255	.3743	.1607	.3205	.1685
C-193	.2522	.1129	.2177	.1481
C-167	.4323	.1849	.3835	.1956

## Discussion

Hormones regulating dysfunctional responses of the HPA-axis to chronic stress have been implied in the etiology of most disorders that can be subsumed under the construct of MSD [53–56]. In addition, the role of leptin and its influence on the HPA-axis and its role with painful disorders have been investigated. In our study, we characterized female patients with MSD in comparison with healthy controls with regards to the methylation status of the leptin promoter region. We focused on female subjects as women are known to have a higher prevalence of MSD [57, 58] because methylation patterns were found to be gender-dependent in genome-wide association studies [59]. We performed a methylation analysis of the leptin promoter region that revealed significant negative correlations between methylation at C-289, C-255, C-193, C-167 and leptin levels in female patients, i.e., less methylation is correlated with higher leptin levels. This is plausible as these CpGs are located at binding sites for transcription factors and higher methylation is often associated with repressive effects on gene expression [60]. Transcription factors Sp1 and c/EBPalpha whose binding is favored in states of reduced methylation and increases transcription of the gene upon binding to DNA [61–64].

After only observing significantly lower methylation in CpG-289 in female patients further analysis revealed that in patients fulfilling strict criteria for CWP had significantly lower methylation levels at CpGs -289, -255 and methylation cluster while -167 trended toward significance (*p* = 0.09) It also serves as further support of our interpretation that lower methylation levels facilitate binding of activating transcription factors Sp1 and c/EBPalpha resulting in higher leptin levels. Previous studies have demonstrated similar findings in psychiatric patients suffering from addiction [43]. Thus, in patients with MSD methylation at C-289 being significantly lower can be contributing to observed elevated leptin levels as this is a known binding site for c/EBPalpha. The lack of significant differences in CpG -255 and the methylation cluster could be attributed to lower pain burden in these patients compared with MSD patients suffering distinctly from CWP. This is plausible as self-reported pain has been shown to be associated with leptin levels [31]. Further significant hypomethylation in CpG -255 and methylation cluster could be a likely corollary, especially as this is a known binding site of Sp1.

Higher leptin levels in patients with painful conditions are biologically plausible as previous study demonstrated increased leptin levels in patients with FMS [28, 65] despite other studies showing an opposite effect [32, 66]. Leptin also plays a crucial role in the development of neuropathic pain in animal models of nerve injury [40, 67] and has been demonstrated to cause allodynia and hyperalgesia [42] (which are hallmarks of neuropathic pain conditions but also of central sensitization and nociplastic pain). The observation is congruent with the fact that a subset of patients with FMS shows signs of small fiber neuropathy [10, 11]. Similar findings have been previously shown in patients with FMS where BMI and elevated leptin levels are independently associated with self-reported pain [31]. Chronic stress is known to cause a dysregulation of the stress response as mediated by the HPA-axis [68]; here leptin has been found to play a significant role as well [69–73] . Taken together our current findings and the fact that leptin levels are significantly higher in these female patients with MSD (publication under review) confer a plausible interrelational connection with leptin regulation in patients with MSD, especially with CWP.

One of the limitations of our and other epigenetic studies is the utilization of DNA from whole blood cells for analysis. It has been shown that different tissues demonstrate similar methylation levels [74], other cases have reported tissue-specific levels [75], whereas neuronal tissue is preferable, most study designs don’t allow for it being readily available. A further limitation is the lack of data on how many possible participants declined to take part in the study after positive eligibility screening as well as on location of recruitment (support group, Pain Clinic, Department of Psychosomatics and Psychotherapy). A potential for a degree of self-selection bias is however mitigated by stringent selection criteria that led to a study population with a high disease burden.

In conclusion, to our knowledge, this is the first study to thoroughly investigate a large collective of patients with MSD and pain as the leading symptom with regards to the epigenetic regulation of leptin expression. Our study demonstrated that transcriptional regulation is in part regulated through methylation on an epigenetic level. Future studies should further validate our results of site-specific promoter methylation of patients compared to controls and increased methylation stratified by degree of widespread pain and stress levels.

## Supplementary Information

**Additional file 1: Table S1.** Leptin primer list.

**Additional file 2: Fig. S1.** Methylation levels (mean ± 95% CI) of all CpGs in female patients and female controls.

## Data Availability

The datasets used and/or analyzed during the current study are available from the corresponding author on reasonable request.
