# Supplementary figures and images for: Leptin promoter methylation in female patients with painful multisomatoform disorder and chronic widespread pain
Source: Clin Epigenetics. 2022 Jan 21;14:13. doi: 10.1186/s13148-022-01235-5 (PMC8783406; doi:10.1186/s13148-022-01235-5)

Fig. S1

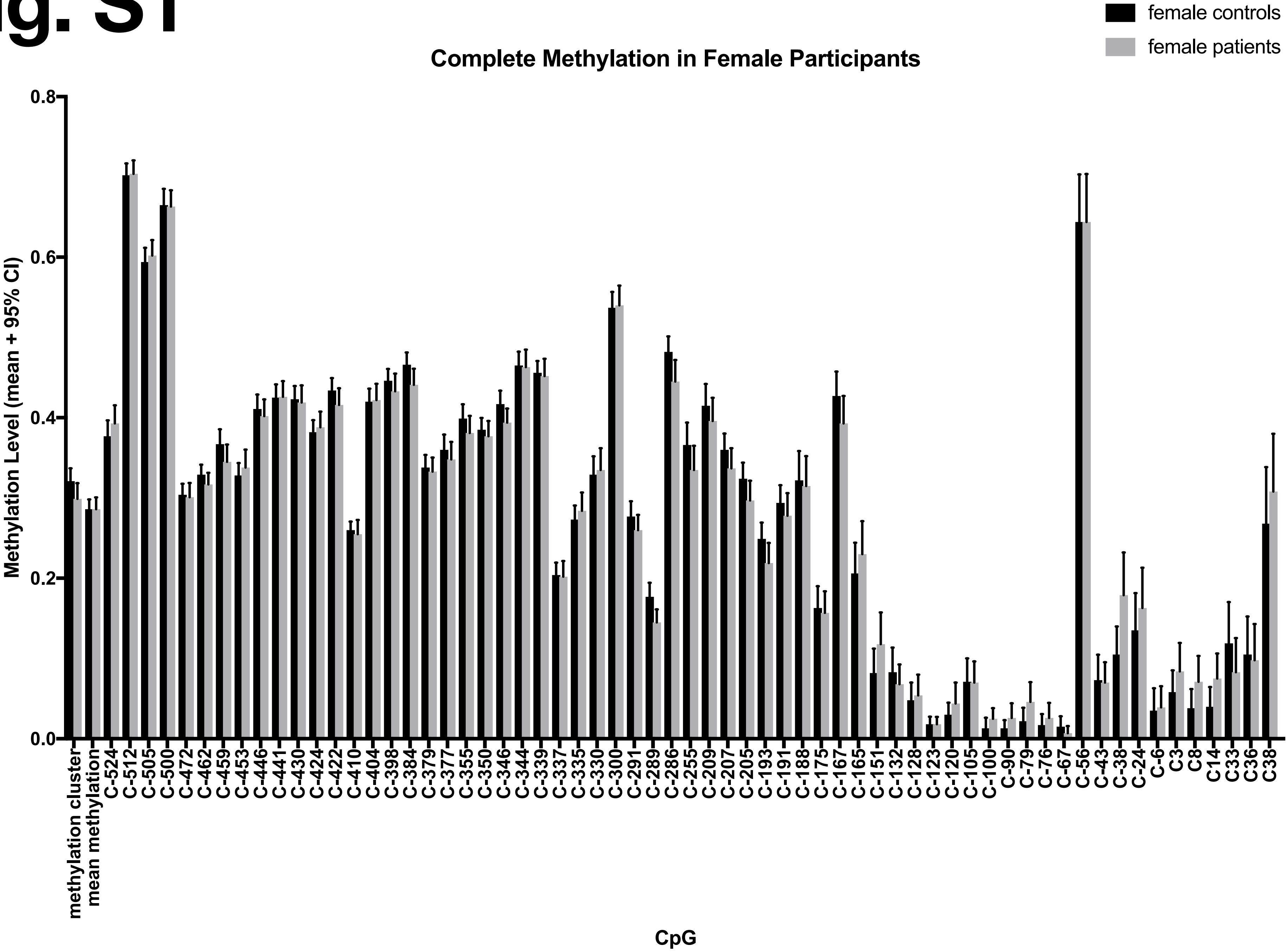

Supplement: Supplementary file 2 — Additional file 2: Fig. S1. Methylation levels (mean ± 95% CI) of all CpGs in female patients and female controls. [file 13148_2022_1235_MOESM2_ESM.pdf]
